# Acute Dilatation of the Stomach

**Published:** 1908-04-18

**Authors:** 


### Medicine.

### ACUTE DILATATION OF THE STOMACH.

Acute overdistension of the stomach, quite apart from excessive ingestion of foods or drinks, is a well-recognised complication both of acute diseases such as typhoid fever, scarlet fever, lobar pneumonia, and pericarditis, and of certain sub-acute or chronic illnesses and cachexias. It is also met with not infrequently as a serious sequela after surgical operations. It is commoner after intra-abdominal operations than after others, it is true; but the following examples succeeded operations in which the peritoneum was not opened.

Reynier records the case of an anæmic woman who had had kolpo-perineorrhaphy performed six years previously for retroversion of the uterus, and now came in for nephropexy. The peritoneum was not interfered with. The anæsthetic used was chloroform, and there was little or no vomiting either during its administration or immediately after it. A striking degree of asthenia for which no adequate cause could be found persisted for several days afterwards, and the bowels could not be made to act in spite of the administration both of purgatives and of enemata. On the fifth day the condition was alarming. There was no sign of anything being wrong at the site of operation. The most obvious point noted was that the abdomen was markedly distended, with an impaired percussion note in the centre and a tympanitic note in the flanks. The impaired note extended from above to some distance below the umbilicus, where a large and obviously fluctuating tumour, not unlike an ovarian cyst, could be felt. Laparotomy seemed the only thing that could save the patient's life, and the tumour turned out to be nothing else than an enormously distended stomach which reached very nearly to the pubes. The stomach gave an impaired percussion note because it was full of fluid, and it had pushed aside the small intestine into the flanks. Nearly fourteen pints of thick brownish material were let out upon puncturing the stomach wall.

Great relief resulted for the time being, but in two days' time the extreme gastrectasis had recurred, and the patient was so weak that she died.

An instance in which similar acute dilatation of the stomach followed operation upon a limb is recorded by Morris in the Pathological Society's Transactions. The patient was a weakly man, aged 37, who required amputation of the right foot for tuberculous mischief. His appetite and digestion seemed quite good, and the sequel was not expected. The anæsthesia was begun with ether and continued with chloroform, lasting twenty minutes altogether. An hour later excessive vomiting set in, extraordinary quantities of a greenish fluid, free from smell, being ejected from the stomach. Intestinal obstruction was closely simulated. The vomiting resisted every effort that was made to relieve it, and death occurred forty-eight hours after the operation. At the autopsy the stomach was enormously dilated and filled nearly the whole abdomen. The intestines were compressed behind it, and they were contracted and empty. Nothing else was abnormal. A similar case was recorded by Schnitzler. A student, aged 17, was operated upon for bilateral genu valgum. Two days after the original operation chloroform was given again for the purpose of applying plaster of Paris bandages. On coming round the lad was exceedingly sick, and for three days he continued to vomit great quantities of yellowish-green fluid, which was never feculent. The bowels could not be made to act. All food by the mouth was stopped, but the vomiting continued. No excessive distension of the abdomen was noted, though the gastric region became rather full. On the third day death ensued from exhaustion. At the autopsy the stomach and the first part of the duodenum were both enormously dilated, and there was no other abnormality.

Many other instances could be quoted of this fatal gastrectasis after operations elsewhere than upon the abdomen. Albrecht, for instance, mentions two women who died from this cause after amputation of the breast and resection of the elbow respectively. In each case there had been no previous symptoms of disgestive disorder.

Successful treatment clearly depends upon early diagnosis, followed at once by lavage of the stomach and interdiction of even fluids by the mouth. As regards the ætiology of the condition various theories have been propounded. Some have thought that a real obstruction arises, either from tetanic contraction of the pylorus or from a twisting of the bowel at the time of operation; but neither of these theories can be maintained in view of the facts that in some cases the pylorus and duodenum are as widely dilated as is the stomach, and that in others the operation has had nothing to do with the abdomen. Mechanical interference with the solar plexus by traction upon the viscera and by manipulations during the operation may be an additional factor in the cases that arise after laparotomies.

Neither is it easy to explain the gastrectasis upon any theory of nervous reflex. If the dilatation arose only after operations upon the abdomen, reflex nervous influences might well be invoked; but when precisely similar trouble may result from operations upon the foot, the knee, the breast, or the elbow, something more than a simple reflex must be at work. This is not to deny nervous influence altogether, for in all probability the gastrectasis is nervous; not only the musculature of the stomach, but also the vasomotor and secretory functions are out of order. What, then, leads to the acute motor, vasomotor, and secretory paralyses? The most probable explanation seems to be that they are the after-effects of the anæsthetic; and it is worthy of note that in almost every instance chloroform has been the anæsthetic used. In certain patients it seems to paralyse the solar plexus.

The cases that have been published are naturally those which have been most severe, and nearly all of these have been fatal. Besides these, however, there are very large numbers of others in which the symptoms are similar, but less terrible in degree.

